# Surviving in Mountain Climate Refugia: New Insights from the Genetic Diversity and Structure of the Relict Shrub *Myrtus nivellei* (Myrtaceae) in the Sahara Desert

**DOI:** 10.1371/journal.pone.0073795

**Published:** 2013-09-18

**Authors:** Jérémy Migliore, Alex Baumel, Marianick Juin, Bruno Fady, Anne Roig, Nathalie Duong, Frédéric Médail

**Affiliations:** 1 Institut Méditerranéen de Biodiversité et d’Ecologie marine et continentale (IMBE), Aix-Marseille Université, UMR CNRS 7263/IRD 237/Avignon Université, Campus Aix - Technopôle de l’Environnement Arbois-Méditerranée, Aix-en-Provence, France; 2 INRA, UR 629, Ecologie des Forêts Méditerranéennes (URFM), Site Agroparc - Domaine Saint Paul, Avignon, France; University of Lausanne, Switzerland

## Abstract

The identification of past glacial refugia has become a key topic for conservation under environmental change, since they contribute importantly to shaping current patterns of biodiversity. However, little attention has been paid so far to interglacial refugia despite their key role for the survival of relict species currently occurring in climate refugia. Here, we focus on the genetic consequences of range contraction on the relict populations of the evergreen shrub *Myrtus nivellei*, endemic in the Saharan mountains since at least the end of the last Green Sahara period, around 5.5 ka B.P. Multilocus genotypes (nuclear microsatellites and AFLP) were obtained from 215 individuals collected from 23 wadis (temporary rivers) in the three main mountain ranges in southern Algeria (the Hoggar, Tassili n’Ajjer and Tassili n’Immidir ranges). Identical genotypes were found in several plants growing far apart within the same wadis, a pattern taken as evidence of clonality. Multivariate analyses and Bayesian clustering revealed that genetic diversity was mainly structured among the mountain ranges, while low isolation by distance was observed within each mountain range. The range contraction induced by the last episode of aridification has likely increased the genetic isolation of the populations of *M. nivellei*, without greatly affecting the genetic diversity of the species as a whole. The pattern of genetic diversity observed here suggests that high connectivity may have prevailed during humid periods, which is consistent with recent paleoenvironmental reconstructions.

## Introduction

During millennia under various successive periods of climatic changes, some areas have facilitated the survival of biota and species; these refugia have attracted increasing attention in biodiversity studies [1]–[3]. Together with fossil data, studies based on DNA polymorphism have revealed the unexpected diversity of glacial refugia (e.g. [4]–[5]). Ranging from peninsular macrorefugia to cryptic microrefugia and nunataks, they have undoubtedly contributed to structuring the current patterns of biodiversity [6]–[9]. However, the question of how individual species persist when they are confined to climate refugia remains open [3]. In this context, extant interglacial refugia provide opportunities to solve this question, since they can be studied directly *in situ* using a wide range of evolutionary ecology methods, whereas the exact locations and areas occupied in the past by glacial refugia often are uncertain [10]–[11]. The genetic structure of relict populations surviving at present in climate refugia after severe range contraction events should therefore provide key information about the evolutionary consequences of climate change and related ecological processes such as fragmentation [12]–[14]. Several studies have been carried out on these lines in temperate and tropical regions ([15]–[17], for example), but there is little knowledge about the extent of current refugia in arid and semi-arid regions [1], [18], even if they cover 41% of the earth’s whole land surface [19]. Therefore, we have focused our study on the largest desert worldwide, the Sahara desert (*ca.* 9 million km^2^), which acts either as a barrier or a corridor between tropical Africa and temperate regions [20].

**Figure 1:**
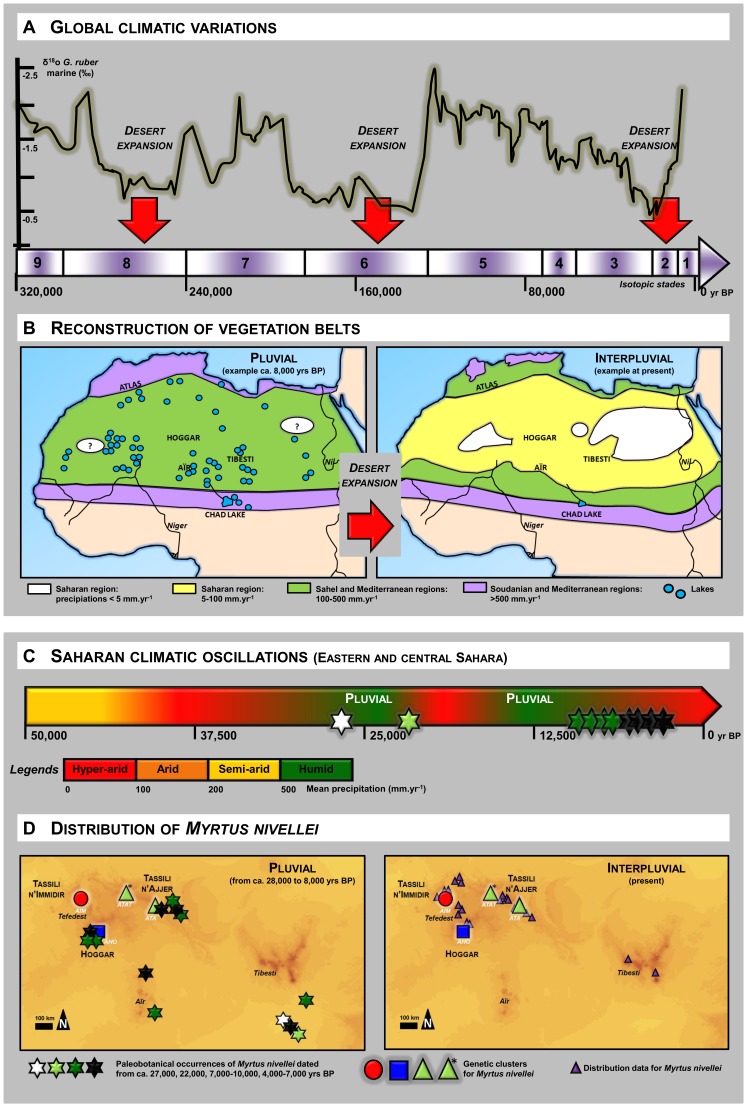
Climatic oscillations in the Sahara and distribution of *Myrtus nivellei*. Global climatic oscillations (A) in relation with the patterns of distribution of vegetation belts (B) (modified from [23], [87]–[88]) and impact of Saharan climatic oscillations with the alternation of pluvial (humid) and interpluvial (arid) periods (C) on the distribution of *Myrtus nivellei* (D). Each individual is denoted by a four-letter code: one letter for the country (A for Algeria), two letters for the mountain range (TA for Tassili n’Ajjer, HO for Hoggar and IM for Immidir), and one letter for the wadi sampled (see Figure S1 as supporting information).

Since the onset of desert conditions in the Sahara at least 7–10 Ma B.P. during the late Pliocene [21], the region has been subjected to a series of sharply contrasting humid and arid periods, known as “pluvials” and “interpluvials”, triggered by orbital (Milankovitch) cycles, monsoon circulation variability and feedbacks of vegetation cover changes on regional climate ([22]–[23]; Figure 1A). During humid phases, the Sahara was a patchwork of savannahs, megalakes and swampy areas abounding with wildlife of all kinds (Figure 1B). But after the early Holocene African Humid Period, from around 5.5 ka B.P. onwards, a process of desertification led to the disappearance of the “Green Sahara” and its dense hydrological and lacustrine network (Figure 1B) [24]–[25]. The main effects of the most recent desertification event in the Sahara are assumed to have been the great contraction of species distribution ranges and the associated fragmentation of populations, constituting a great challenge for the survival and regeneration of species. Biodiversity declined massively, as evidenced by fish and crocodile populations surviving in widely scattered and constantly dwindling bodies of water in the Sahara [26]–[27]. Plants suffered the same fate, and species such as the pluri-millennial *Cupressus dupreziana* are confined to extremely restricted areas (the last specimens occur in an area of only 700 km^2^ [28]).

Several lines of evidence indicate that some moist habitats restricted to the Hoggar, Tassili n’Ajjer, Tassili n’Immidir, Tefedest, Aïr and Tibesti ranges may constitute climate refugia *sensu* Hampe *et al.* [3]. At mid altitudes (between 1500 and 2500 m a.s.l.), these Saharan mountains offer sufficiently high levels of humidity and relatively cool temperatures favoring the survival of species not adapted to the drier and hotter conditions prevailing in the surrounding extremely arid lowlands [29]–[31].

The occurrence of a relict Mediterranean flora (*Myrtus nivellei, Cupressus dupreziana*, *Pistacia atlantica, Nerium oleander, Ephedra altissima, Lavandula antinea, Salvia chudaei*, etc.) in the mountainous central Saharan climate refugia has probably resulted from a sudden drastic environmental change that occurred at the periphery of the Mediterranean biome [32]–[33]. These climate relicts constitute a unique but under-studied biological system for predicting the responses of Mediterranean plants to the increasing warming and aridification under climate change [34]–[35]. There is only limited information about the genetic diversity of Saharan climate relicts [36], although there is some evidence that the Sahara has played a major role in shaping biogeographic patterns by acting as a powerful driver of speciation and evolutionary changes [20], [37]. Among other questions, it has not yet been established whether these Saharan mountain ranges sheltered populations in the past from strong processes of demographic decline and genetic erosion during desertification events. Empirical knowledge about these changes could be used to validate simulations of the consequences of range contractions on molecular diversity [38] which have suggested that loss of genetic diversity depended both on the rate of change and the generation times of species.

In the present study, we examine the genetic structure of the endemic Nivelle myrtle (*Myrtus nivellei* Batt. et Trab.), the only species growing in the central Saharan mountains (in four Algerian mountain ranges: the Hoggar, Tassili n’Ajjer, Tefedest, and Tassili n’Immidir ranges, and in Chad: the Tibesti mountains [39]), among the 5650 Myrtaceae species described in the world [40]. The current populations, which are highly variable in size - ranging from less than ten to several hundreds of individuals - are scattered over small and sparse areas located in rocky, sandy wadis (the beds or valleys of streams which are usually dry except during occasional rainy episodes) or near permanent water points, from 1400 to 2500 m a.s.l. *Myrtus nivellei* is closely related with *Myrtus communis* L., and is expected to have diverged from that species only since the colonization of the Saharan mountains [41]. Saharan populations have therefore been affected by natural range contraction processes, originating from the circum-Mediterranean *M. communis* after at least three independent seed-mediated migration events and isolation in the Saharan mountains [41].

As fossil and molecular data combined with paleoecological reconstructions (Figure 1) support the occurrence of a strong range contraction during the late Pleistocene, our study aims at better understanding to what extent the levels of genetic diversity of the relict *M. nivellei* have been affected by past climatic changes. Indeed, range contractions, shifts and expansions can leave a lasting imprint on molecular diversity [38]. To detect their effect on the current distribution of *M. nivellei*, this study investigates how the current genetic diversity of *M. nivellei* is distributed within the Saharan climate refugia after the successive climate changes. According to an extensive sampling of *M. nivellei,* we adopted a multi-marker approach combining highly variable microsatellite and Amplified Fragment Length Polymorphism markers (AFLP; [42]) to examine multiple loci distributed throughout the genome.

## Materials and Methods

### Biological Model and Sampling Strategy

*Myrtus nivellei* is an endemic shrub, 1–2 m high, found exclusively in central Saharan mountains, where rainfall varies between 50 and 100 mm per year [32], [39], [43]–[45]. It is morphologically similar to the Mediterranean *Myrtus communis* but has more linear leaves [46]. The fruit is a small globose blue-black berry 1 cm in diameter. Its white flowers are pollinated by insects and its seeds are dispersed by animals. The plant has been reported by several botanists to flower at different times of the year, presumably in response to irregular rainfalls events [44].

During field trips conducted between 2005 and 2009 in the three main Algerian mountain ranges (the Hoggar, Tassili n’Ajjer, and Tassili n’Immidir ranges), 167 samples of *M. nivellei* were collected from 23 wadis for the analysis of the overall genetic structure in the study area (see Figure 1D and Figure S1 as supporting information; permission issued by the Institut National de Recherche Forestière in Algeria). In the Tassili n’Immidir, a more intensive sampling campaign was undertaken in order to study the pattern of genetic diversity at a finer scale, hence the addition of 48 individuals sampled along three kilometers of the Tarit wadi. In total 215 individuals were sampled in central Sahara. As botanical exploration is challenging in the Saharan mountains, and because of the great patchiness of local myrtle populations, it is likely that our population sampling was not exhaustive. However, it covers the main currently known populations. As the myrtle is a resprouter shrub, leaves were collected only from physically isolated individuals growing several meters apart, to avoid collecting leaves from the same individual genotype (genet).

### DNA Extraction

The fresh leaves of *M. nivellei* were dried in silica gel and stored at −20°C. Total DNA was extracted from silica gel-dried samples (30 mg of leaves), using the Doyle and Doyle procedure [47], with the following modifications: 1.4 mM NaCl, 20 mM EDTA, 100 mM Tris-HCl pH 8 and 4% of Hexadecyltrimethyl-ammonium bromide. DNA concentration and quality were determined using a photometer (Biophotometer Eppendorf, Hamburg, Germany).

### Microsatellite Genotyping

Eleven nuclear microsatellite markers (*Myrcom*1 to *Myrcom*11) developed for *Myrtus communis* by Albaladejo *et al.* [48] were used in this study. Polymerase chain reactions were performed in 50-µL solutions containing 250 ng DNA, buffer, 3 mM MgCl_2_, 0.6 mM dNTPs, 0.2 µM of each primer, and 2.5 units of *Taq* polymerase (MP Biomedicals, Illkirch, France) in a *PTC-200 Gradient Thermal Cycler* (MJ Research, Waltham, Massachusetts, USA), using touchdown PCR protocols [48]. The 5′ ends of the forward primers were labelled with *FAM*, *HEX*, *TAMRA* or *ATTO565* with a view to multiplexing the PCR products. The fluorescently-labelled PCR products were separated by capillary electrophoresis with a 500 bp size standard (*LIZ500(*−*250)*), using an *ABI Prism® 3730xl* (Applied Biosystems, FosterCity, California, USA) automatic sequencer. Alleles were sized using the GeneMapper version 4.1 software program (Applied Biosystems). The microsatellite dataset was deposited in Dryad (doi:10.5061/dryad.1vt02).

### AFLP Genotyping

The Amplified Fragment Length Polymorphism reaction was performed as described by Vos *et al.* [42] with slight modifications: 500 ng DNA was digested for 3 h at 37°C with 10 units of *Eco*RI (Eurofins MWG, Ebersberg, Germany) and for 3 h at 65°C with 4 units of *Tru*9I (Eurofins MWG) in a total volume of 25 µL. Digestion products were then ligated for 3 h at room temperature by adding 2.5 pmol *Eco*RI and 25 pmol *Mse*I adaptors, 0.5 units T4 DNA ligase and 10 mM ATP (Eurofins MWG). Five µL of 8-fold diluted ligation products were used as a template in the preamplification step, using 10 pmol of *Eco*RI (+A) and *Mse*I (+C) primers, 0.16 mM dNTPs, 0.65 mM MgCl_2_ and 1.5 units of *Taq* DNA polymerase (MP Biomedicals) in a final volume of 50 µL. The preamplification thermocycle profile was 94°C for 2 min, followed by 20 cycles at 94°C for 45 s, 56°C for 45 s, 72°C for 1 min and 72°C for 10 min. Selective amplification was performed using 5 pmol of *Eco*RI (+ANNN) and *Mse*I (+CNNN) primers with 5 µL of 100-fold diluted preamplification product in a final volume of 20 µL. Each selective amplification reaction mixture contained 0.5 mM dNTPs, 0.65 mM MgCl_2_ and 1 unit of *Taq* DNA polymerase (MP Biomedicals). The selective amplification thermocycle profile was: 94°C for 2 min, 10 touchdown cycles of 94°C for 30 s, 65°C for 30 s (step −0.7°C per cycle), 72°C for 1 min, followed by 20 cycles at 94°C for 30 s, 56°C for 30 s, 72°C for 1 min and 72°C for 5 min (thermocycler: MJ Research PTC-200). After screening selective primers, three primer combinations giving clear-cut profiles were chosen for further analysis (*Eco*RI-AAGG with *Mse*I-CCAG, *Eco*RI-AAC with *Mse*I-CAC, *Eco*RI-AAC with *Mse*I-CAA).

Polyacrylamide electrophoreses (0.4%) were performed on a 96-capillary Megabace 1000 automated sequencer (Amersham Biosciences Europe GmbH, Freiburg, Germany). Markers were scored in view of the fluorescent peaks in the chromatograms after calibration with a standard size marker (*ETROX900*), using the *Genetic Profiler* software program (Amersham Biosciences). Low quality profiles (giving peaks with a low signal intensity of generally less than 50 relative fluorescent units) were discarded and manual scoring was carefully performed in order to detect any markers prone to errors not giving reliable peaks. The reproducibility and reliability of the AFLP markers were checked by repeating the complete analysis from the DNA amplification to the AFLP screening on 20 samples for each pair of primers [49]. Some of the markers were not reproducible, and therefore a genotyping error rate of 5.7% was estimated. The AFLP dataset was deposited in Dryad (doi:10.5061/dryad.1vt02).

### Estimation and Distribution of Genetic Diversity

To determine that identical genotypes were clones, the probability that a zygote would acquire a given diploid genotype *P_gen_* was calculated as described by Parks and Werth [50] using SSR data. This gave *P_gen_* = (Π *p_i_*) 2*^h^*, where *p_i_* is the frequency in the population of each allele present in the genotypes and *h* is the number of heterozygous loci. The *P_gen_* value gives thus the probability that two different samples will have the same genotype by chance. The *P_gen_* was calculated using the GenAlEx 6.4 software program [51]. When clonality was detected, only a single ramet per genet was kept in all the genetic analyses both for microsatellites and AFLPs.

Genetic diversity at microsatellite markers was characterized by Shannon’s information index *I*, the observed heterozygosity *H_o_*, the unbiased expected heterozygosity *UH_e_* and the Wright *F*-statistics (*F_ST_* and *F_IS_*), using the GenAlEx 6.4 program [51]. We also used the rarefaction approach implemented in the HP-Rare program [52] to estimate the allelic richness (*AR*) and the private allelic richness (*PAR*) after standardizing to the smallest population sample size (26).

### Genetic Differentiation, Clustering Analyses and Isolation by Distance

To investigate the genetic structure of *M. nivellei* within and among mountain ranges, all analyses were performed in parallel on microsatellite and AFLP datasets. Hierarchical analyses of the molecular variance (AMOVA; [53]) were conducted using GenAlEx 6.4 program [51] and significance of the *Ф_PT_*-statistics was tested using 10000 random permutations.

A discriminant analysis of principal components (DAPC) was then conducted using AMOVA results to identify groups of individuals as priors. The DAPC multivariate method relies thus on data transformation using principal component analysis as a prior step to discriminant analysis, in order to explore genetic diversity without any explicit evolutionary models and underlying assumptions (R2.15.0: adegenet package; R Development Core Team) [54].

In parallel, *M. nivellei* individuals were also assigned to genetically homogeneous clusters via the model-based clustering algorithm provided in Structure 2.3.3 program [55]–[57]. The Bayesian analysis was run under the admixture and recessive allele models after a burn-in period of 100000 and 1000000 simulations for *K* from 2 to 23 and 5 iterations for each *K*-value. The most likely number of clusters was determined using the log-probability of the data (*lnPr(X/K)*; [55]) and the method of Evanno *et al.* [58].

Finally, to test for isolation by distance [59], the Mantel test implemented in GenAlEx 6.4 [51] was performed, adopting the null hypothesis that the geographic and genetic distances were not correlated. In each mountain, pairwise genetic differences between wadis were measured using *Ф_PT_*-statistics. In the thoroughly sampled Tarit wadi, the distances between genotypes were calculated as described in GenAlEx 6.4 [51] in the case of dominant and codominant markers.

## Results

### Evidence of Clonal Growth and Levels of Polymorphism

Among the eleven microsatellite loci developed for *M. communis* [48], seven loci were polymorphic in *M. nivellei* and were therefore used to estimate genetic diversity parameters. The total number of alleles ranged between 5 and 13 alleles, with a mean value of 7 alleles per locus (Table S1). Microsatellite data showed that several individuals shared the same genotype: out of the 167 *M. nivellei* individuals analyzed, there were only 117 different genetic profiles with generally one or two ramets. The probability of obtaining the same genetic profile (*P_gen_*) was extremely low (ranging from 2.7×10^−3^ in the Hoggar, to 3.5×10^−3^ in the Tassili n’Immidir, and 8.7×10^−4^ in the Tassili n’Ajjer; see Table S2). Two identical genotypes were therefore assumed to be clones. Most of the clones were observed in the same wadi network, growing some tens to hundreds of meters apart. The largest distances between ramets belonging to the same genet were observed in the Tassili n’Immidir and Tassili n’Ajjer, where values of up to 2.5–3.9 km and 9.7–19.0 km were recorded, respectively. Tassili n’Ajjer was characterized by the largest proportion of clones (31.4%), as compared with 30.9% in the Tassili n’Immidir and 25.7% in the Hoggar. In the more intensively sampled Tarit wadi (Immidir), the proportion of clones observed reached 14.5% (*n* = 69). Clones detected by microsatellites were not rejected by AFLP data, since the small amount of observed variation from AFLP markers between individuals having identical microsatellite genotypes fell within the bounds of scoring errors for AFLPs (5.7%) or could be explained by somatic mutations in some of the clones as explained by Bakker *et al.* [60].

The microsatellite markers showed that the lowest levels of allelic richness and private allelic richness (after rarefaction) appeared in the Hoggar (2.38 and 0.43, respectively), as compared with the Tassili n’Immidir (*AR* = 3.49 and *PAR* = 1.65) and Tassili n’Ajjer samples (*AR* = 4.19 and *PAR* = 2.33) (Table 1). However, the highest observed heterozygosity values were obtained in the Hoggar (0.312), and were lower (0.241 on average) in the Tassili n’Ajjer and Tassili n’Immidir. The highest Shannon’s information index was obtained in the Tassili n’Ajjer (0.832) and the lowest in the Hoggar (0.617).

**Table 1:** Genetic diversity of *Myrtus nivellei.*

Regions studied	*N*	*AR*	*PAR*	*H_o_*	*UH_e_*	*I*	*F_IS_*
**Hoggar**	26	2.38	0.43	0.312 (0.062)	0.420 (0.065)	0.617 (0.090)	0.075 (0.100)
**Tassili n’Immidir**	56	3.49	1.65	0.240 (0.052)	0.377 (0.056)	0.711 (0.095)	0.067 (0.129)
**Tassili n’Ajjer**	35	4.19	2.33	0.241 (0.051)	0.448 (0.055)	0.832 (0.102)	0.048 (0.191)
***Mean***	*39*	*3.35*	*1.47*	*0.264 (0.031)*	*0.415 (0.033)*	*0.720 (0.056)*	*0.063*

Genetic characteristics of *Myrtus nivellei* in each of the three central Saharan mountain ranges (Hoggar, Tassili n’Immidir and Tassili n’Ajjer). Microsatellite markers were used to determine the total number of individuals (*N*), the allelic richness (*AR*) and the private allelic richness after rarefaction (*PAR*), the observed heterozygosity (*H_o_*), the unbiased expected heterozygosity (*UH_e_*), the Shannon’s information index (*I*) and the *F_IS_*-statistic.

The AFLP analysis yielded 119 scored fragments. Their length ranged from 66.3 to 453.1 bp in the three pairs of primers analyzed. Seventy-seven bands were counted in the Hoggar, 83 in the Tassili n’Immidir and 87 in the Tassili n’Ajjer. More private bands were counted in the samples from the Tassili n’Ajjer (14) than in those from Hoggar (8) and Tassili n’Immidir (9).

### Among-mountain Genetic Structure

The AMOVA highlighted the key structuring role of each mountain range: highly significant *Ф_PT_* values were obtained with both AFLPs and microsatellites (0.353 and 0.514, respectively; Table 2). In addition, pairwise *Ф_PT_* distances (Table 3) showed higher genetic differentiation between the Tassili n’Immidir and Tassili n’Ajjer than between the Tassilis and the Hoggar mountains. The *F_ST_* value obtained on the basis of the microsatellite data was 0.314±0.079.

**Table 2:** Genetic differentiation of *Myrtus nivellei.*

Source		*Degree of* *freedom*	*Sum of* *squares*	*Variance* *components*	*Percentage of variance (%)*	*Ф_PT_ statistics*
**Hoggar (microsatellites)**	*Among wadis*	3	20.740	0.552	13	0.131
	*Within wadis*	22	80.183	3.645	87	
**Hoggar (AFLP)**	*Among wadis*	3	24.308	0.865	23	0.225
	*Within wadis*	22	65.500	2.977	77	
**Tassili n’Immidir (microsatellites)**	*Among wadis*	6	44.154	0.620	17	0.166
	*Within wadis*	47	146.624	3.120	83	
**Tassili n’Immidir (AFLP)**	*Among wadis*	6	30.259	0.504	24	0.240
	*Within wadis*	47	75.167	1.599	76	
**Tassili n’Ajjer (microsatellites)**	*Among wadis*	8	71.343	1.546	32	0.319
	*Within wadis*	25	82.539	3.302	68	
**Tassili n’Ajjer (AFLP)**	*Among wadis*	8	40.259	0.496	13	0.133
	*Within wadis*	25	80.800	3.232	87	
**Whole sampling (microsatellites)**	*Among mountains*	2	327.99	4.325	51	0.514
	*Within mountains*	114	466.591	4.095	49	
**Whole sampling (AFLP)**	*Among mountains*	2	120.236	1.549	35	0.353
	*Within mountains*	114	324.226	2.844	65	

Results of the analyses of molecular variance (AMOVA) on microsatellite and AFLP markers for samples of *Myrtus nivellei* were analyzed for each central Saharan mountain range.

**Table 3:** Genetic distances within the distribution range of *Myrtus nivellei.*

	Microsatellite data			AFLP data		
Pairwise *Ф_PT_* distances	Hoggar	Tassili n’Immidir	Tassili n’Ajjer	Hoggar	Tassili n’Immidir	Tassili n’Ajjer
**Hoggar**	0			0		
**Tassili n’Immidir**	0.510	0		0.375	0	
**Tassili n’Ajjer**	0.475	0.534	0	0.241	0.401	0

Pairwise *Ф_PT_* distances between mountain ranges from microsatellite and AFLP markers.

**Figure 2:**
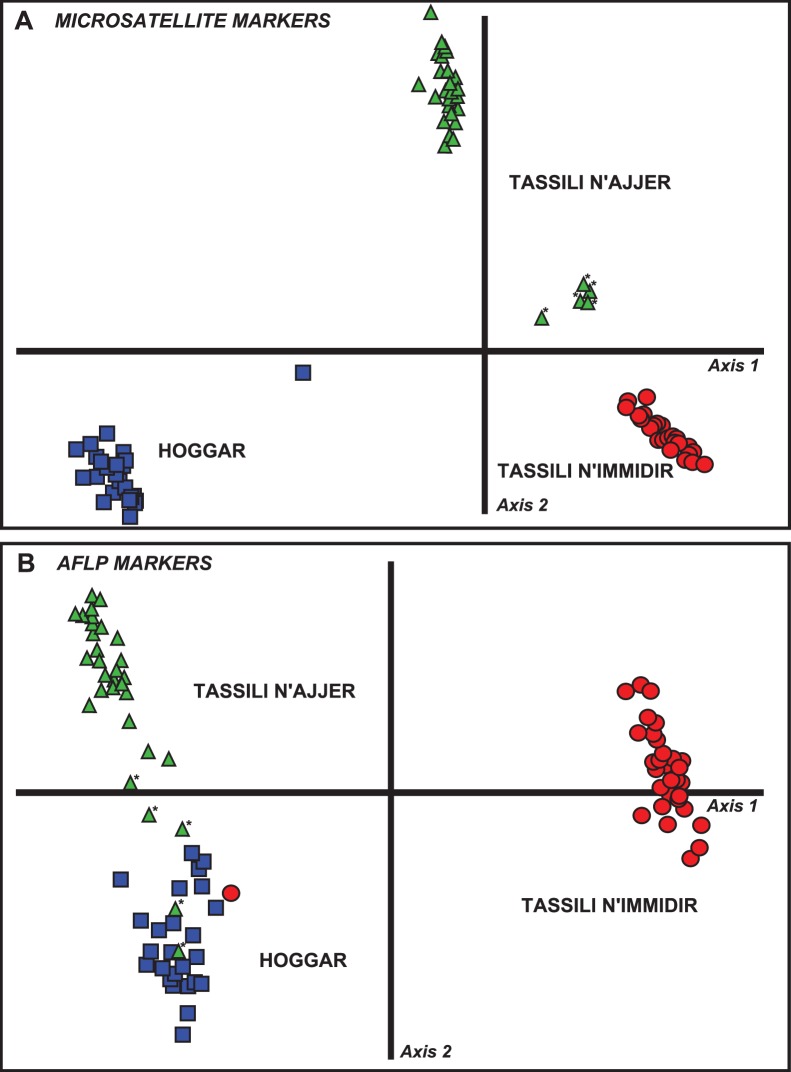
Genetic structure of *Myrtus nivellei* in the central Saharan mountain ranges. DAPC results for microsatellite (A) and AFLP markers (B). Blue squares represent samples from the Hoggar, red circle refer to samples from Tassili n’Immidir, green triangles indicate samples from Tassili n’Ajjer, and asterisks report samples from the ATAT population collected in the northern part of the Tassili n’Ajjer mountain range.

The DAPC and Structure Bayesian analyses yielded similar genetic structures, whether they were based on microsatellite data (Figures 2A, S2A), or on AFLP results (Figures 2B, S2B). The genetic clusters obtained corresponded to the three mountain ranges investigated. However, in the Tassili n’Ajjer, the northernmost samples (ATAT) collected in the Tasedjebest plateau differed from all other samples from the same mountain range (ATAA to ATAI). Indeed, ATAT samples were partially assigned to the Hoggar by AFLP markers, but, were in an intermediate position according to microsatellite data (Figure 2).

### Within-mountain Genetic Structure

Overall, our results revealed a lack of clustering of *M. nivellei* genotypes within each of the three central Sahara mountain ranges investigated. For each mountain range, a significant (*P*<0.01) relationship was observed in the Mantel analysis between the genetic and geographic distances, but very low isolation-by-distance values were obtained. After removing the ATAT samples from the analysis because of their genetic and geographic isolation (Figure 2), the Mantel *R^2^* ranged between 0.013 in the Tassili n’Immidir and 0.077 in the Tassili n’Ajjer based on the microsatellite data, and between 0.020 in the Tassili n’Ajjer and 0.166 in the Hoggar based on the AFLP markers.

The microsatellite data yielded high *F_ST_* values among wadis within mountains. Among wadis, *F_ST_* values decreased from 0.466±0.050 in the Tassili n’Immidir to 0.417±0.060 in the Tassili n’Ajjer and 0.160±0.051 in the Hoggar. Microsatellite data were also used to determine *F_IS_* values, which increased from 0.048±0.191 in the Tassili n’Ajjer to 0.067±0.129 in the Tassili n’Immidir and 0.075±0.100 in the Hoggar (Table 1).

Finally, genetic diversity at microsatellites was particularly low in the Tarit wadi (*H_o_* = 0.229±0.061, *I* = 0.629±0.131) and heterozygote deficiency particularly high (*F* = 0.274±0.066). Neither microsatellites nor AFLPs could detect any particular spatial genetic structure within the Tarit wadi. The Mantel test was not significant (*R^2^* = 0.006, *P* = 0.415) and no clear-cut spatial clustering of genotypes was observed.

## Discussion

### Surviving under Desert Conditions: Evidence of Clonality

There is a surprising high rate of occurrence of clones (30%) in *M. nivellei*, since no repeated clonal reproduction process has so far been evidenced in the genus *Myrtus*. It cannot be ruled out that a process of asexual multiplication may have occurred in *M. nivellei* via asexually produced seeds (apomixis) rather than a process of vegetative fragmentation. However, almost all the *M. nivellei* genets consisted of a very small number of ramets distributed in the same wadi only some tens of meters apart. It therefore seems likely that episodes of catastrophic floods resulting in large amounts of suddenly accumulated rainwater in the dendritic network of wadis may have dispersed the vegetative parts of the myrtle shrubs over large distances.

The exact contribution of clonal reproduction processes to the persistence of *Myrtus* in the Sahara desert still remains to be established. Clonal growth, often combined with longevity [61], can greatly favor the persistence of plants in stressful environments or when drastic environmental conditions prevent the occurrence of sexual reproduction [62]–[67]. During several field campaigns, we found very little or no evidence of sexual reproduction and fructification in *M. nivellei* (F. Médail, personal observations), in sharp contrast with *M. communis* which produces many berries throughout its Mediterranean range. Asexual reproduction is not a rare occurrence in central Saharan tree populations. For example, in *Olea europaea* subsp. *laperrinei*, clones were observed in significant proportions, reaching 26% in the Tassili n’Ajjer, 14% in the Hoggar and between 13 and 40% in the Aïr range [68]–[69]. In the relict tree *Cupressus dupreziana*, clonality via paternal apomixis from the production of unreduced pollen germinating in the seed tissues of a surrogate mother [70] was found to be the only way of reproduction and was considered as a key mechanism to maintain diversity and survive under extreme adverse conditions.

### Extreme Genetic Isolation of *M. nivellei* Populations in the Central Saharan Mountains

Paleoecological studies have shown that central Saharan mountains have repeatedly undergone severe climatic changes from hyper-arid to humid periods during at least 2.5 Ma, concomitantly with the succession of glacial and interglacial periods in the northern hemisphere (Figure 1A). One key aspect of these oscillations was the great steepness of the changes (Figure 1B), which have caused a drastic contraction of the distribution range of *M. nivellei* (Figure 1C, D), as currently observed. Fossil records clearly show the presence of *M. nivellei* in a much larger range of distribution (up to the Chad Basin), and at lower altitudes (*ca.* 300 m a.s.l.) during several Pleistocene and Holocene periods ([71]; Figure 1D).

Our molecular data are congruent with the fossil records. First, a previous study using cpDNA indicated the presence of *Myrtus* in the Saharan mountains since at least the middle Pleistocene [41]. Secondly, the use of nrDNA sequences and the comparisons between microsatellite and AFLP genotypes of *M. nivellei* and *M. communis* clearly show that the two species diverged recently from a common ancestor (J. Migliore, unpublished data). These patterns of genomic divergence are consistent with the hypothesis that *M. nivellei* survived several climatic changes from pluvial to interpluvial periods. The data obtained here using a multimarker approach provide therefore the phylogeographical framework required to examine the effects of fast, drastic climatic changes on genetic diversity and the role played by the central Saharan mountains as climate refugia for relict species [3].

*Myrtus nivellei* populations display a marked genetic isolation within each Saharan mountain range. A high level of genetic differentiation was found among the mountains in the study area (*F_ST_* = 0.314 based on the microsatellite data and *Φ_PT_* = 0.353 based on the AFLP data: see also Table 2). The microsatellite-based pattern of differentiation observed here is comparable to that obtained using nine nuclear SSR loci for the Saharan endemic *Olea europaea* subsp. *laperrinei*, a fairly similar species in terms of its ecology and dispersal strategy. In the case of Laperrine’s olive, the *F_ST_* values ranged from 0.021 to 0.116 between pairs of mountains in southern Algeria and Niger [72], whereas higher *F_ST_* values were obtained here on *M. nivellei* using seven SSR loci, ranging from 0.249 to 0.278 between pairs of Algerian mountains. This higher genetic differentiation in *Myrtus* than in *Olea* may be due to different gene dispersal capacities of these species. In particular, pollen-mediated gene flow over long distance (>2 km) has been reported in the Laperrine’s olive and may maintain connectivity between distant patches, but to our knowledge no data is available regarding pollen dispersal in *Myrtus*.

Based on the molecular clock-dated phylogeographic data obtained on the genus *Myrtus* [41], at least three founder events appear to have occurred from the Mediterranean to the Sahara. This process was suggested by the presence of several cpDNA haplotypes descending from distinct *M. communis* lineages in each of the main central Saharan mountain ranges. Separate populations still inhabit the Hoggar, Tassili n’Ajjer and Tassili n’Immidir nowadays, as established here from multivariate and Bayesian clustering analyses results (Figures 2, S2), and from the amount of private SSR alleles and AFLP bands (Table 1). The private markers detected using three distinct molecular methods (cpDNA sequencing, microsatellite data and AFLP markers) support the existence of three significant evolutionary units [73]–[74] originating from independent episodes of southward colonization by Mediterranean populations [41]. The repeated range contraction processes which occurred did not erase the imprints of the Mediterranean origins of *M. nivellei*.

After the founder events, the combined effects of the geographic distances between mountains (which are located *ca.* 300 to 500 km apart) and the presence of harsh desert barriers during arid periods have probably prevented, or at least greatly restricted, the occurrence of gene flow between mountain ranges, increasing genetic differentiation. By contrast, along the Mediterranean coast, *M. communis* has been less exposed to isolation and several periods of extensive expansion have been described [41]. Although the reduced size and fragmentation of populations can be the main factor affecting the gene flow dynamics occurring among myrtle populations in the central Sahara, another factor could be also the scarcity in pollinators and seed-dispersers. Seeds may have been dispersed over long distances by birds across this large desert [75], which would explain the current patchy patterns of distribution of some Saharan plants [76], most of the trans-Saharan migratory birds are waterfowl, and they mainly disperse plants associated with aquatic habitats [77]–[78]. In addition, only 10% of the passerines in the African-Palearctic migration system have a stopover in the desert [79], and migratory birds avoid high altitude ranges where *M. nivellei* grows (P. Bruneau de Miré, personal communication). The central Saharan mountains can therefore be said to constitute terrestrial “mountain-islands” for Nivelle’s myrtle, since they play the role of true biogeographic islands [80]–[81] or “inselbergs” [30].

### Have Relict Species Persisting in Climate Refugia Maintained their Ability to Expand?

One key question about the relict species which are currently restricted to small areas focuses on whether they would still be able to expand if more favorable climatic conditions occurred [82]. One way of predicting this process is to search for the signatures of past gene flows between remote populations due to expansion events during suitable humid periods. The low level of divergence of the nuclear ribosomal DNA sequences suggest that a process of wide range connectivity or long distance gene flow may have occurred [41]. Here, the difficulty to assign some individuals, especially from the northern Tassili n’Ajjer ATAT (Figure 2), supports the idea that ancient gene flows may have occurred between populations belonging to separate mountain ranges, as also evidenced by Bayesian clustering analyses (Figure S2). The pairwise differentiation indices calculated here per mountain range (Table 3) suggest that the Hoggar played a key role by connecting the populations of the Tassili n’Ajjer and Tassili n’Immidir, as previously reported in a study on the genetic structure of the Laperrine’s olive [72]. The lower level of isolation of the Hoggar was also confirmed by the distribution of nrDNA haplotypes determined from previously sequenced ITS and ETS data [41]: two private nrDNA haplotypes were found to be restricted to the Tassili n’Immidir and to the Tassili n’Ajjer, and no private haplotype was detected in the Hoggar. Secondary contacts between genetically differentiated mountain range populations during Saharan pluvials may have led to the homogenization of the *M. nivellei* nrDNA sequences in the Hoggar.

Although high to moderate levels of genetic differentiation were detected among mountain ranges (Table 2 and *F_ST_* values), there is no support for the existence of clear genetic clusters within mountains among wadis. This low spatial genetic structure was also observed on the finest scale of analysis in the Tarit wadi, which was intensively sampled in the Tassili n’Immidir. To account for these results, which are in contrast with the high level of population fragmentation, it seems likely that a higher degree of landscape connectivity occurred during past humid periods. *Myrtus nivellei* probably persisted *in situ* during arid periods in local mountainous climate refugia and recolonized lowland areas during humid periods. In Saharan mesophilous species, range expansion is known to increase gene flow between populations connected by suitably moist habitats, e.g. via the huge network of rivers that existed during humid periods [21], [83]–[84]. By contrast, increased aridity resulting from dry periods may have induced considerable range contraction, resulting in the complete disappearance of the lowland populations and the current survival of only some very isolated and patchy populations in the highlands.

The genetic diversity of *M. nivellei* (Table 1) is considerably lower than that of two *M. communis* populations from the Guadalquivir river in southern Spain (*H_o_* = 0.482 and *H_e_* = 0.624, on average, using the same loci [48]). However, we observed that *M. nivellei* has conserved a significant part of its genetic diversity, because both microsatellite and AFLP markers detected significant levels of polymorphism in all populations. We can suggest that this shrub has been able to efficiently track its suitable habitats during range contractions, *i.e.* without experiencing severe demographic bottlenecks (see for *M. communis*: [85]–[86]). Our results obtained in a hyper-arid biome support the fact that a long generation time associated with a resprouting strategy and vegetative reproduction is a crucial compensatory factor slowing down the decrease of genetic diversity, as already suggested for relict rain forest trees [82]. Population dynamics simulations also indicate that species with long generation times suffer less from range contraction, especially in the case of fast contraction [38].

Overall, our study of *M. nivellei* in the central Saharan mountains provides several new insights on the extremely strong persistence of biodiversity in climate refugia. In these myrtle Saharan populations, the most obvious consequence of habitat and range contraction processes is an extreme population fragmentation, which, unexpectedly, has not resulted in extreme genetic erosion. The persistence of this endemic plant in the central Saharan mountains may have been punctuated in the past by several periods of expansion with high population connectivity, as shown by the imprints left in the myrtle genetic diversity. Comparative phylogeographic studies on some other rare Mediterranean species restricted to the central Saharan mountains are therefore required in order to establish whether these species will be able to migrate and contribute to the next “green Sahara” period, or on the contrary, to survive future drastic aridification events.

## Supporting Information

Figure S1 **Sampled populations of** ***Myrtus nivellei*** **within each of the central Saharan mountain ranges (Hoggar, Tassili n’Immidir and Tassili n’Ajjer).** Blue squares represent samples from the Hoggar, red circle refer to samples from Tassili n’Immidir, green triangles indicate samples from Tassili n’Ajjer, and asterisks report samples from the ATAT population collected in the northern part of the Tassili n’Ajjer mountain range. DIVA-GIS software was used (http://www.diva-gis.org/).
(TIF)

Figure S2 **Genetic structure of** ***Myrtus nivellei*** **based on Bayesian clustering (Structure) for microsatellite (** ***K*** **= 3) and AFLP markers (** ***K*** **= 4).** Blue squares represent samples from the Hoggar, red circle refer to samples from Tassili n’Immidir, green triangles indicate samples from Tassili n’Ajjer, and asterisks report samples from the ATAT population collected in the northern part of the Tassili n’Ajjer mountain range.
(TIF)

Table S1 **Genetic characteristics for each microsatellite locus (** ***MYRCOM1*** **to** ***MYRCOM11*** **) examined for** ***Myrtus nivellei*** **.** The total number of alleles, the allelic richness (*AR*), the observed heterozygosity (*H_o_*), the unbiased expected heterozygosity (*UH_e_*), and the Shannon's information index (*I*) were calculated for each microsatellite marker.
(DOCX)

Table S2 **Probabilities of occurrence of a diploid genotype for each individual of** ***Myrtus nivellei*** **analyzed through microsatellite markers in the Hoggar, Tassili n’Immidir and Tassili n’Ajjer.**
(DOCX)
