# Supplementary figures and images for: Surviving in Mountain Climate Refugia: New Insights from the Genetic Diversity and Structure of the Relict Shrub Myrtus nivellei (Myrtaceae) in the Sahara Desert
Source: PLoS One. 2013 Sep 18;8(9):e73795. doi: 10.1371/journal.pone.0073795 (PMC3776782; doi:10.1371/journal.pone.0073795)

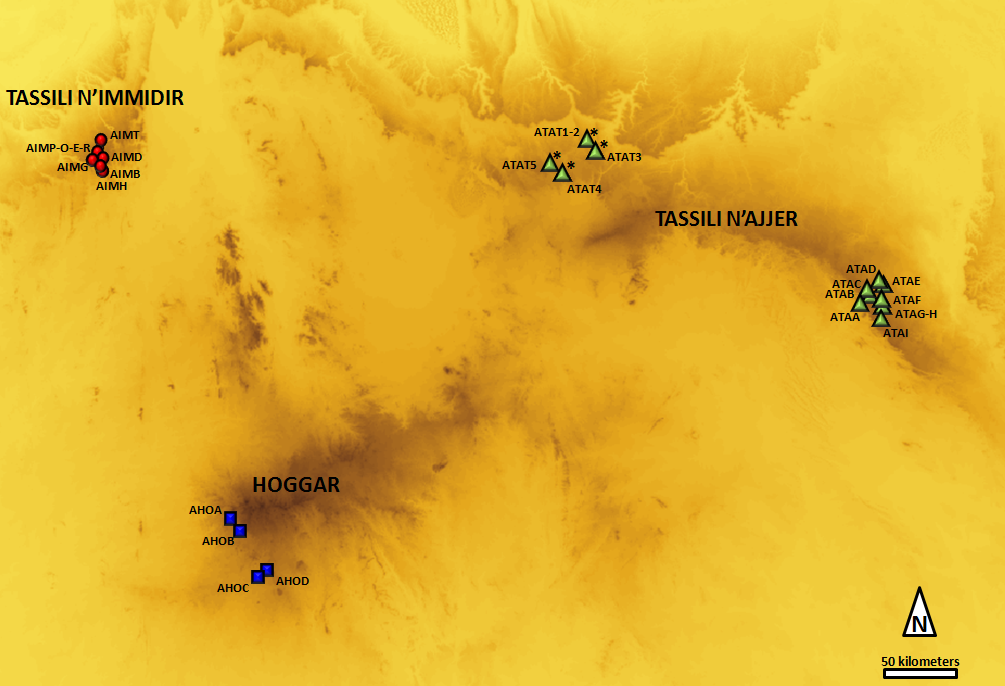

Supplement: Figure S1 — Sampled populations of Myrtus nivellei within each of the central Saharan mountain ranges (Hoggar, Tassili n’Immidir and Tassili n’Ajjer). Blue squares represent samples from the Hoggar, red circle refer to samples from Tassili n’Immidir, green triangles indicate samples from Tassili n’Ajjer, and asterisks report samples from the ATAT population collected in the northern part of the Tassili n’Ajjer mountain range. DIVA-GIS software was used (http://www.diva-gis.org/). (TIF) [file pone.0073795.s001.tif]

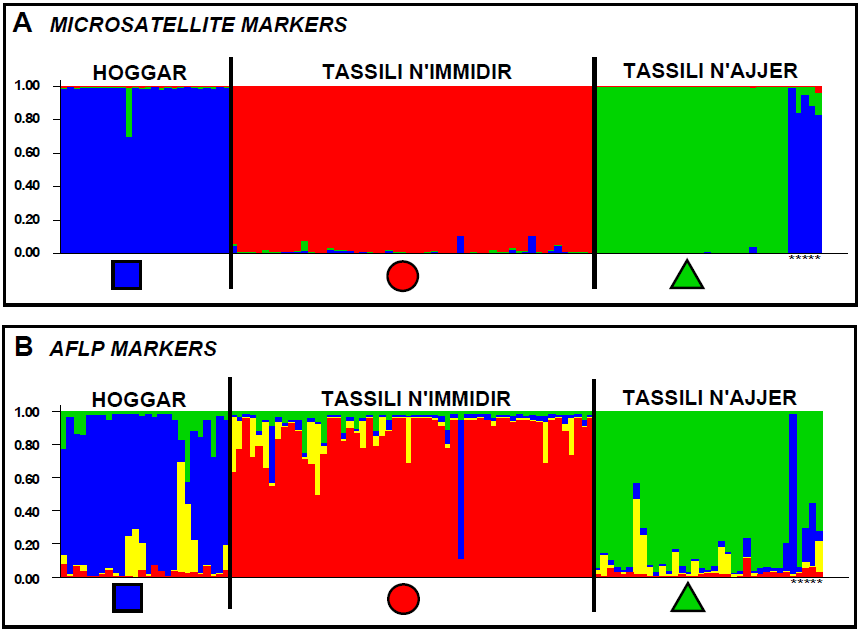

Supplement: Figure S2 — Genetic structure of Myrtus nivellei based on Bayesian clustering (Structure) for microsatellite ( K = 3) and AFLP markers ( K = 4). Blue squares represent samples from the Hoggar, red circle refer to samples from Tassili n’Immidir, green triangles indicate samples from Tassili n’Ajjer, and asterisks report samples from the ATAT population collected in the northern part of the Tassili n’Ajjer mountain range. (TIF) [file pone.0073795.s002.tif]
