# Supplementary material for: Surviving in Mountain Climate Refugia: New Insights from the Genetic Diversity and Structure of the Relict Shrub Myrtus nivellei (Myrtaceae) in the Sahara Desert
Source: PLoS One. 2013 Sep 18;8(9):e73795. doi: 10.1371/journal.pone.0073795 (PMC3776782; doi:10.1371/journal.pone.0073795)
Supplement: Table S1 — Genetic characteristics for each microsatellite locus ( MYRCOM1 to MYRCOM11 ) examined for Myrtus nivellei . The total number of alleles, the allelic richness (AR), the observed heterozygosity (Ho), the unbiased expected heterozygosity (UHe), and the Shannon's information index (I) were calculated for each microsatellite marker. (DOCX) [file pone.0073795.s003.docx]

**Table S1. Genetic characteristics for each microsatellite locus (*MYRCOM1* to *MYRCOM11*) examined for *Myrtus nivellei*.**

| **Microsatellite markers** | **Number of alleles** | **AR** | ***H_o_*** | ***UH_e_*** | ***I*** |
| --- | --- | --- | --- | --- | --- |
| ***MYRCOM1*** | Monomorphic | / | / | / | / |
| ***MYRCOM2*** | 5 | 3.000 (0.577) | 0.215 (0.070) | 0.373 (0.094) | 0.602 (0.126) |
| ***MYRCOM3*** | 5 | 3.000 (0.577) | 0.235 (0.117) | 0.369 (0.097) | 0.614 (0.138) |
| ***MYRCOM4*** | Monomorphic | / | / | / | / |
| ***MYRCOM5*** | 5 | 2.333 (0.333) | 0.448 (0.025) | 0.523 (0.045) | 0.780 (0.117) |
| ***MYRCOM6*** | Monomorphic | / | / | / | / |
| ***MYRCOM7*** | 7 | 5.000 (0.577) | 0.215 (0.025) | 0.503 (0.065) | 0.941 (0.108) |
| ***MYRCOM8*** | 13 | 5.667 (1.856) | 0.313 (0.095) | 0.464 (0.044) | 0.884 (0.122) |
| ***MYRCOM9*** | Monomorphic | / | / | / | / |
| ***MYRCOM10*** | 11 | 4.000 (1.528) | 0.110 (0.040) | 0.204 (0.098) | 0.454 (0.222) |
| ***MYRCOM11*** | 5 | 3.333 (0.667) | 0.314 (0.057) | 0.469 (0.060) | 0.764 (0.082) |
| ***Mean*** |  | *3.762 (0.408)* | *0.264 (0.031)* | *0.415 (0.033)* | *0.720 (0.056)* |

The total number of alleles, the allelic richness (*AR*), the observed heterozygosity (*H_o_*), the unbiased expected heterozygosity (*UH_e_*), and the Shannon's information index (*I*) were calculated for each microsatellite marker.
