# Supplementary material for: Surviving in Mountain Climate Refugia: New Insights from the Genetic Diversity and Structure of the Relict Shrub Myrtus nivellei (Myrtaceae) in the Sahara Desert
Source: PLoS One. 2013 Sep 18;8(9):e73795. doi: 10.1371/journal.pone.0073795 (PMC3776782; doi:10.1371/journal.pone.0073795)
Supplement: Table S2 — Probabilities of occurrence of a diploid genotype for each individual of Myrtus nivellei analyzed through microsatellite markers in the Hoggar, Tassili n’Immidir and Tassili n’Ajjer. (DOCX) [file pone.0073795.s004.docx]

**Table S2. Probabilities of occurrence of a diploid genotype for each individual of *Myrtus nivellei* analyzed through microsatellite markers in the Hoggar, Tassili n’Immidir and Tassili n’Ajjer.**

| **Samples Hoggar** | ***Pgen*** |  | **Samples Tassili n'Immidir** | ***Pgen*** |  | **Samples Tassili n'Ajjer** | ***Pgen*** |
| --- | --- | --- | --- | --- | --- | --- | --- |
| AHOA02 | 2.2E-03 |  | AIMG02 | 1.4E-08 |  | ATAA03 | 6.4E-06 |
| AHOA04 | 9.7E-04 |  | AIMP02 | 2.4E-04 |  | ATAA04 | 3.4E-03 |
| AHOA05 | 5.8E-03 |  | AIMP06 | 2.7E-02 |  | ATAB03 | 7.0E-04 |
| AHOA06 | 1.2E-02 |  | AIMP08 | 2.2E-03 |  | ATAB06 | 1.3E-04 |
| AHOA07 | 4.6E-05 |  | AIMP09 | 1.2E-03 |  | ATAB07 | 8.7E-05 |
| AHOA09 | 1.2E-03 |  | AIMP11 | 3.0E-07 |  | ATAB08 | 1.5E-03 |
| AHOB01 | 1.5E-03 |  | AIDE01 | 6.7E-05 |  | ATAC01 | 8.7E-04 |
| AHOB02 | 4.9E-03 |  | AIDE02 | 4.3E-04 |  | ATAC03 | 1.7E-03 |
| AHOB03 | 6.8E-03 |  | AIMH01 | 8.6E-06 |  | ATAC04 | 3.9E-04 |
| AHOB04 | 2.7E-03 |  | AIMH04 | 7.8E-05 |  | ATAD07 | 3.2E-04 |
| AHOB05 | 2.6E-05 |  | AIMB01 | 7.9E-04 |  | ATAD08 | 1.2E-03 |
| AHOB06 | 1.2E-04 |  | AIMB02 | 8.8E-08 |  | ATAE01 | 6.2E-03 |
| AHOB09 | 2.4E-03 |  | AIMB03 | 1.3E-03 |  | ATAE02 | 9.4E-05 |
| AHOB10 | 3.8E-04 |  | AIMB04 | 2.7E-06 |  | ATAF01 | 2.5E-04 |
| AHOB11 | 3.1E-03 |  | AIMB05 | 5.7E-07 |  | ATAF02 | 6.5E-04 |
| AHOB12 | 1.8E-05 |  | AIMB06 | 3.2E-05 |  | ATAF03 | 1.2E-03 |
| AHOB13 | 3.3E-03 |  | AIMB07 | 1.4E-03 |  | ATAG01 | 7.3E-04 |
| AHOB14 | 7.2E-03 |  | AIMB09 | 4.1E-04 |  | ATAG02 | 3.6E-05 |
| AHOC01 | 4.0E-03 |  | AIMB10 | 7.0E-05 |  | ATAG03 | 3.0E-06 |
| AHOC05 | 3.1E-03 |  | AIMB11 | 1.4E-04 |  | ATAG04 | 3.0E-03 |
| AHOC06 | 4.3E-03 |  | AIMB12 | 3.8E-03 |  | ATAG05 | 6.8E-05 |
| AHOD02 | 1.1E-04 |  | AIME02 | 2.4E-06 |  | ATAG06 | 2.7E-04 |
| AHOD03 | 1.8E-04 |  | AIMO09 | 4.7E-04 |  | ATAG07 | 1.7E-03 |
| AHOD04 | 3.3E-03 |  | AIMO11 | 1.2E-06 |  | ATAG09 | 4.9E-05 |
| AHOD05 | 3.3E-04 |  | AIMR01 | 7.5E-07 |  | ATAG10 | 1.7E-03 |
| AHOD06 | 2.3E-04 |  | AIMR02 | 2.9E-05 |  | ATAH03 | 4.2E-04 |
|  |  |  | AIMR03 | 2.2E-05 |  | ATAI01 | 4.4E-08 |
|  |  |  | AIMR06 | 1.7E-02 |  | ATAI02 | 1.2E-04 |
|  |  |  | AIMR07 | 3.1E-03 |  | ATAI03 | 3.3E-05 |
|  |  |  | AIMR10 | 2.0E-05 |  | ATAI04 | 3.7E-03 |
|  |  |  | AIMR11 | 3.5E-03 |  | ATAT01 | 4.2E-13 |
|  |  |  | AIMR12 | 5.5E-03 |  | ATAT02 | 4.2E-09 |
|  |  |  | AIMR13 | 2.3E-05 |  | ATAT03 | 2.2E-09 |
|  |  |  | AIMR14 | 1.5E-02 |  | ATAT04 | 3.1E-12 |
|  |  |  | AIMR15 | 5.6E-04 |  | ATAT05 | 4.1E-11 |
|  |  |  | AIMR16 | 1.1E-05 |  |  |  |
|  |  |  | AIMTA01 | 3.8E-02 |  |  |  |
|  |  |  | AIMTA02 | 1.6E-02 |  |  |  |
|  |  |  | AIMTB02 | 5.3E-04 |  |  |  |
|  |  |  | AIMTC01 | 1.5E-02 |  |  |  |
|  |  |  | AIMTC05 | 1.8E-04 |  |  |  |
|  |  |  | AIMTC10 | 8.8E-04 |  |  |  |
|  |  |  | AIMTD01 | 6.5E-03 |  |  |  |
|  |  |  | AIMTD05 | 2.3E-04 |  |  |  |
|  |  |  | AIMTD10 | 1.5E-04 |  |  |  |
|  |  |  | AIMTE01 | 7.7E-04 |  |  |  |
|  |  |  | AIMTE05 | 6.4E-06 |  |  |  |
|  |  |  | AIMTF01 | 7.6E-06 |  |  |  |
|  |  |  | AIMTF08 | 1.2E-02 |  |  |  |
|  |  |  | AIMTF16 | 7.5E-05 |  |  |  |
|  |  |  | AIMTG01 | 9.1E-06 |  |  |  |
|  |  |  | AIMTG05 | 3.3E-04 |  |  |  |
|  |  |  | AIMTG11 | 1.3E-02 |  |  |  |
|  |  |  | AIMTH01 | 1.6E-05 |  |  |  |
|  |  |  | AIMTH07 | 3.1E-03 |  |  |  |
|  |  |  | AIMTH14 | 2.5E-03 |  |  |  |
| *Mean* | *2.7E-03* |  | *Mean* | *3.5E-03* |  | *Mean* | *8.7E-04* |
